# Environmental Gradients Shape Mammal and Galliform Bird Communities in a Mountain Reserve Through Species Turnover and Niche Differentiation

**DOI:** 10.3390/biology15090672

**Published:** 2026-04-24

**Authors:** Qinlong Dai, Yunqiao Zhang, Liuyang He, Jiahao Zhang, Lifeng Zhu, Qiang Dai

**Affiliations:** 1 School of Medicine, Nanjing University of Chinese Medicine, Nanjing 210023, China; qinlongdai@163.com; 2 Key Laboratory of National Forestry and Grassland Administration on Biodiversity Conservation on the Qinghai-Xizang Plateau, Chengdu Institute of Biology, CAS, Chengdu 610213, China; zhangyq@cib.ac.cn (Y.Z.); zhangjiahao0307@163.com (J.Z.); 3 Liziping Giant Panda’s Ecology and Conservation Observation and Research Station of Sichuan Province, Nanchong 637000, China; liuyanghe2026@163.com; 4 Shimian Management and Protection Station, Giant Panda National Park, Ya’an 625400, China; 5 University of Chinese Academy of Sciences, Beijing 101408, China

**Keywords:** camera trapping, community organization, environmental heterogeneity, niche differentiation, turnover, human disturbance

## Abstract

Protected areas are often treated as internally homogeneous conservation units, yet their communities may be structured either as discrete modules or as continuous gradients shaped by environmental heterogeneity and human disturbance. Using camera-trap data from Liziping Nature Reserve, China, we examined the spatial organization of mammal and galliform bird communities and tested whether species-level environmental responses help explain community structure. From 109 camera-trap sites surveyed between October 2017 and July 2020, we obtained 6688 independent detections and retained 17 species for analysis. We combined β-diversity decomposition, clustering, NMDS ordination, single-species occupancy models, clustering of environmental response coefficients, and Mantel tests. Community variation was dominated by turnover rather than nestedness, and clustering based on co-occurrence and relative activity patterns did not reveal well-separated discrete modules. Instead, NMDS indicated continuous variation along environmental gradients, with elevation and vegetation productivity as the strongest correlates. Occupancy models showed marked species-specific environmental responses, especially to elevation, habitat structure, and human disturbance, and β-based clustering suggested two broad environmental response groups. Although human influences did not affect all species uniformly, some species showed clear sensitivity to recent disturbance and human-modified landscapes. These results indicate that communities in Liziping are better characterized as continuous gradient structures than as discrete modules, and suggest that conservation should emphasize the maintenance of environmental heterogeneity, habitat continuity, connectivity, and differentiated management of human activities within mountain protected areas.

## Simple Summary

Biodiversity is not evenly distributed even within protected areas, so understanding where different animals occur within a reserve is important for effective conservation. We studied the distribution of mammals and ground-living birds in Liziping Nature Reserve, China, using camera traps at 109 sites. From more than 6000 independent records, we examined how animal communities varied across the reserve and how these variations were related to environmental conditions and human activities. We found that biodiversity within the reserve was not concentrated in just a few fixed community types. Instead, species changed across the landscape as conditions such as elevation, vegetation, habitat features, and human disturbance changed. Different species also responded to these conditions in different ways, suggesting that many species can live in the same reserve because they use different habitats and resources. This means that conservation in mountain protected areas should not focus only on a few hotspots. It should also protect environmental variety, habitat connections, and small habitat features across the whole landscape. Although human activities did not affect all species equally, some species were clearly sensitive, indicating that grazing and other disturbances still require careful management.

## 1. Introduction

Since the Anthropocene [1], human impacts on ecosystems have intensified, while global biodiversity has undergone persistent decline [2]. Many researchers have suggested that the Earth has already entered a sixth mass extinction [3,4]. Against this background, maintaining biodiversity and designing effective conservation strategies have become increasingly complex [5]. Although protected areas are widely regarded as the core spatial units for biodiversity conservation [6,7], they are not internally homogeneous “safe habitats.” Instead, they usually encompass complex gradients shaped by environmental factors such as elevation, vegetation, and climate, while also being continuously influenced by multiple forms of human activity, including roads, settlements, grazing, poaching, collecting, and tourism [8,9,10]. Therefore, treating protected areas simply as relatively uniform conservation units is no longer sufficient to explain the spatial variation of biodiversity within them. In contrast, greater attention to the internal organization of communities within protected areas and their relationships with environmental gradients, as well as to how natural environmental heterogeneity and human disturbance jointly drive such spatial differentiation, is essential for understanding the mechanisms that maintain multispecies coexistence and for improving conservation management [11,12,13].

Community composition and its spatial variation along environmental gradients are closely linked to how biodiversity is maintained within protected areas and to the priorities of conservation management [14,15,16]. A key question is whether community composition and species associations are organized as clearly bounded discrete modules or instead vary continuously along environmental gradients. If communities are structured into relatively independent modules, conservation management may focus more on identifying and maintaining key habitat units. In such cases, differences among communities are more likely to reflect nestedness, with species-poor communities representing subsets of species-rich communities, making large habitat patches particularly important for conservation in fragmented systems [17,18]. In contrast, if communities primarily vary continuously along environmental gradients, differences among communities are more likely to arise from species turnover. In this case, conservation should place greater emphasis on maintaining environmental heterogeneity, habitat continuity, and connectivity, for example, through multi-scale strategies or complementary protected-area networks across environmental gradients [19,20,21].

Animal communities are often shaped jointly by environmental heterogeneity and human disturbance, a process that is particularly pronounced in mountain protected areas [22]. As elevation and topography change, environmental factors such as vegetation productivity, habitat structure, and hydrothermal conditions vary systematically, potentially leading different species to exhibit differentiated distributions along environmental gradients [23]. At the same time, human activities, including roads, built-up areas, and livestock grazing, continue to influence landscapes both within and around protected areas [24], thereby further altering species distributions and community structure [25]. Therefore, analyses based solely on overall community composition are insufficient to fully reveal the mechanisms underlying multispecies coexistence. It is also necessary to consider species-specific responses to environmental factors and human disturbance, and to examine whether species exhibit differentiated habitat selection and environmental preferences within shared spatial contexts [26,27]. By linking community-level patterns with species-level environmental responses, it becomes possible to more effectively determine whether communities vary continuously along environmental gradients or instead contain groups of species sharing similar ecological preferences [28], thereby providing insights into the mechanisms that maintain multispecies coexistence within protected areas [29].

Liziping Nature Reserve in Sichuan, China, provides an ideal setting for investigating the spatial organization of communities and their underlying drivers in mountain protected areas. The reserve harbors a high diversity of endangered, threatened, and endemic species [30], but conservation efforts have long been centered primarily on the giant panda (*Ailuropoda melanoleuca*) and its habitat [31,32]. At the same time, the reserve contains pronounced environmental gradients, while surrounding agriculture, grazing, and tourism continue to influence its ecological processes [30]. Under these conditions, it remains unclear whether communities within the reserve are organized as discrete modules or instead vary continuously along environmental gradients. This distinction has direct implications for conservation management, determining whether priority should be given to key habitat patches or to the maintenance of environmental heterogeneity and habitat connectivity. Therefore, examining community organization and its drivers at the scale of Liziping Nature Reserve will not only improve understanding of the mechanisms underlying species coexistence in mountain ecosystems but also provide a scientific basis for more refined management of mountain protected areas.

In this study, we used camera-trap data to examine the spatial organization of mammal and galliform bird communities in the Liziping Nature Reserve at two complementary levels. First, using analyses of beta-diversity and community composition, we asked whether community structure across sites is better characterized as discrete modules or as a continuous gradient along environmental variation. Second, using species co-occurrence patterns and occupancy models, we asked whether species differ in their responses to environmental gradients and human disturbance, and whether similarity in species co-occurrence and activity structure is associated with similarity in environmental responses. By linking community-level structure with species-level environmental responses, we aimed to clarify the mechanisms underlying multispecies coexistence in a mountain protected area and to provide a basis for more community-inclusive conservation planning.

## 2. Materials and Methods

### 2.1. Study Area

Liziping Nature Reserve, covering 479.40 km^2^, is located in the Xiaoxiangling Mountains on the eastern edge of the Himalaya–Hengduan Mountains region in Sichuan, China. The reserve is dominated by mid- to high-elevation mountainous terrain, with deeply dissected topography, pronounced vertical relief, and an elevational range of approximately 1330–4551 m [33]. Along this elevational gradient, vegetation transitions from montane evergreen broad-leaved forest and mixed conifer–broadleaf forest to subalpine coniferous forest, subalpine shrubland, and alpine shrub meadow, forming a distinct pattern of vertical vegetation zonation [30].

Liziping Nature Reserve is surrounded by seven villages, with a total population of approximately 6000. Within the reserve boundary, about 60 households are associated with local land use. Agriculture and livestock grazing are the main livelihood activities in the surrounding communities, and tourism is also developed in part of the area. Cropland in the surrounding area totals about 1.33 km^2^, of which 0.007 km^2^ lies within the reserve [34]. There are no designated grazing pastures within the reserve. Instead, livestock grazing is mainly conducted in a free-ranging manner by surrounding communities, and animals can enter forest, shrub, and meadow habitats. Livestock grazing involves all seven surrounding villages, with approximately 2000 cattle, 4000 sheep, 1000 horses, and 2000 yaks. Some livestock regularly enter the reserve, but their exact numbers cannot be fully quantified.

The reserve currently manages human activities mainly through routine patrolling, public outreach and education, and the promotion of alternative livelihoods such as tourism. Grazing activities have been continuously regulated, which has helped maintain overall grazing pressure within the reserve at a relatively low level, but they have not been completely eliminated. As livestock are often managed in a free-ranging manner in surrounding communities, occasional entry into reserve habitats still occurs.

### 2.2. Infrared Camera Deployment

From October 2017 to July 2020, infrared camera traps were deployed throughout the reserve, with a minimum spacing of 800 m between cameras. Cameras were preferentially placed at sites with frequent animal activity, such as mountain trails, areas near water sources, and open sites under forest cover, to maximize detection probability. Each camera was mounted on a tree trunk or other stable substrate at approximately 50 cm above the ground and oriented toward likely animal travel routes. Cameras operated continuously for 24 h per day and, when triggered, recorded three consecutive photographs and one 10 s video, with a 5 s interval between triggers. Consecutive records of the same species at the same camera within 30 min were treated as a single independent event. Camera identity, species identity, and detection time were extracted from each independent record for subsequent analyses.

To quantify variation in species activity across camera-trap sites, we calculated a relative abundance index (RAI) for each species at each camera location. RAI was defined as the number of independent detection events per 100 effective camera-trap days. Independent events were defined using a 30 min interval criterion between consecutive records of the same species at the same camera, and effective camera-trap days were calculated as the total number of days during which each camera was operational. The resulting RAI matrix was used to calculate Bray–Curtis dissimilarities among sites for site-based analyses and, after matrix transposition, among species for species-level analyses. For subsequent community and occupancy analyses, species with fewer than 30 independent detections and recorded at fewer than 10 camera sites were excluded to reduce instability associated with sparse data.

### 2.3. Environmental Data

Land-cover data were obtained from the ESA WorldCover 10 m 2021 v200 dataset [35], which provides global information on the distribution of major land-cover types at a spatial resolution of 10 m. Within the study area, relevant land-cover classes were extracted and the shortest distance from each camera-trap site to patches of different land-cover types was calculated to characterize habitat structure and human-modified environments. Based on the actual distribution of land-cover types in the study area and their ecological relevance, representative and widely distributed classes were selected, including forest (tree), shrubland (shrub), grassland (grass), cropland (crop), built-up areas (built), bare land (bare), and water bodies (water). Distance variables were calculated for each class, resulting in the predictors dist_tree, dist_shrub, dist_grass, dist_crop, dist_built, dist_bare, and dist_water, which were used to represent the potential influence of habitat types and human land use on community distribution patterns. To improve the characterization of aquatic environments, we also incorporated spatial data on rivers and streams provided by the Forestry Department of Sichuan Province (2015) [36]. The final water-distance variable was defined as the minimum distance from each site to either ESA WorldCover water bodies or mapped rivers and streams, thereby improving the completeness and accuracy of water-related spatial information.

Topographic variables were derived from a 30 m resolution digital elevation model obtained from the Geospatial Data Cloud of the Chinese Academy of Sciences (http://www.gscloud.cn). In addition to elevation (ele), terrain variables including slope, northness, terrain ruggedness index (TRI), and topographic wetness index (TWI) were calculated from the elevation raster. All topographic variables were retained at a spatial resolution of 30 m for subsequent analyses.

Vegetation productivity was characterized using the normalized difference vegetation index (NDVI) and enhanced vegetation index (EVI) derived from Landsat 8 surface reflectance imagery (Collection 2 Level-2) available in Google Earth Engine. To represent peak growing-season conditions while reducing the influence of cloud contamination and short-term interannual variability, we first extracted the annual maximum NDVI and EVI values from images acquired during June–September for each year from 2016 to 2020. The resulting annual maximum layers were then combined using a median composite to produce stable estimates of vegetation productivity for subsequent analyses. Cloud, cloud-shadow, and snow pixels were masked using the QA_PIXEL quality band, and surface reflectance bands were rescaled with standard Landsat scaling factors prior to index calculation. NDVI and EVI were calculated as follows:

(1) $$NDVI=\frac{NIR-Red}{NIR+Red}$$

(2) $$\mathrm{EVI}=2.5\times \frac{\mathrm{NIR}-\mathrm{Red}}{\mathrm{NIR}+6\;\mathrm{Red}\;-\;7.5\;\mathrm{Blue}+1}$$

where NIR, Red, and Blue represent the surface reflectance in the near-infrared, red, and blue spectral bands, respectively.

We considered two complementary dimensions of human influence in the analyses. First, relatively stable landscape modification was represented by distance to built-up areas (dist_built) and cropland (dist_crop), derived from land-cover data. These variables capture the long-term spatial footprint of settlements and agricultural land use. Second, recent anthropogenic disturbance (hereafter disturbance) was quantified at each site using a camera-trap-based relative abundance index (RAI) derived from independent detections of humans and livestock. Because grazing in the reserve occurs mainly through free-ranging livestock from surrounding communities rather than within designated pastures, a spatially explicit grazed-area variable was not available for the study period.

All continuous environmental predictors were standardized (mean = 0, SD = 1) prior to model fitting. The quadratic elevation term (ele2) was calculated as the square of the standardized elevation variable to reduce collinearity between linear and quadratic terms.

To reduce multicollinearity among environmental predictors, variance inflation factors (VIFs) were calculated for all candidate variables. Variables with VIF values greater than 5 were considered highly collinear and excluded from subsequent analyses. TRI and EVI were initially included as candidate variables but were removed after collinearity screening. The remaining variables were retained for the occupancy analyses (Figure S1 and Table S1). For NMDS ordination and envfit analyses, environmental variables were fitted individually to the ordination space, so TRI and EVI was retained to characterize vegetation productivity.

### 2.4. Analytical Framework and Data Structures

To distinguish community-level variation among sites from similarity among species, we used two complementary data structures in subsequent analyses. For community-level analyses, we used a site-by-species matrix based on species presence–absence data. For species-level analyses, we used the transposed species-by-site matrix, from which Jaccard dissimilarity was calculated from occurrence data and Bray–Curtis dissimilarity from species-specific RAI across sites.

### 2.5. Beta-Diversity and Null Model Analyses

Community dissimilarity among sites was quantified using Jaccard beta diversity based on species presence–absence data and partitioned into turnover and nestedness components following Baselga (2010) using betapart [37,38]. To test whether the observed level of community differentiation deviated from random expectations, we randomized the community matrix while preserving site richness and species occurrence frequencies using the swap algorithm in vegan [39]. We performed 9999 randomizations and compared the observed mean pairwise Jaccard dissimilarity with the null distribution.

### 2.6. Clustering Analysis

To examine whether species-level co-occurrence and relative activity patterns formed discrete modules, hierarchical clustering analyses were performed on the species-level Jaccard and Bray–Curtis distance matrices. Candidate solutions with k = 2–16 groups were evaluated using mean silhouette width to assess cluster separation. To determine whether the observed species arrangement departed from random expectations, we calculated a Δ statistic, defined as the difference between mean between-group and within-group dissimilarities for a given clustering solution. Larger Δ values indicate stronger differentiation among groups relative to within-group similarity, but do not by themselves imply the presence of well-separated discrete modules. Observed Δ values were compared with a null distribution generated by randomly assigning species to groups while preserving group sizes. Thus, Δ analysis was used to test whether the observed structure was more organized than expected by chance, whereas silhouette width was used to evaluate the strength of cluster separation. Significance was assessed using 9999 randomizations.

### 2.7. NMDS Ordination and Environmental Fitting

NMDS (Non-metric Multidimensional Scaling) ordinations were performed on the species-level Jaccard and Bray–Curtis distance matrices to examine patterns of species co-occurrence and relative activity structure. Analyses were conducted using the metaMDS function in vegan (version 2.7-2) [39], and environmental vectors were fitted with envfit. Each ordination point represented a species rather than a camera-trap site. Correlation strength (r^2^) and significance were assessed using 9999 permutations. Environmental predictors included the variables representing topography, vegetation productivity, habitat structure, hydrological conditions, and human disturbance.

### 2.8. Occupancy Analysis

We used camera-trap data to model species occupancy across the study area. Detection histories were constructed by dividing the monitoring period into 14-day sampling occasions, with each species coded as detected or not detected at each site during each occasion. To examine spatial variation in occupancy, the model included 13 environmental predictors: elevation (ele), elevation squared (ele2), slope (slope), northness (northness), topographic wetness index (twi), vegetation productivity (ndvi), distance to forest (dist_tree), shrubland (dist_shrub), grassland (dist_grass), bare land (dist_bare), cropland (dist_crop), built-up areas (dist_built), and recent anthropogenic disturbance (disturb). Since elevation was standardized prior to analysis and the quadratic term (ele2) was calculated as the square of standardized elevation, the signs of the linear and quadratic elevation coefficients can be used to classify species-specific elevational response curve types (Table S2). Occupancy probability was modeled as

(3) $$logit(\psi_{si})=\alpha_{s}+\sum_{v=1}^{V}\beta_{sv}Z_{iv}$$

where ψ_si_ represents the occupancy probability of species s at site i, α_s_ is the species-specific intercept, β_sv_ denotes the regression coefficient describing the effect of environmental predictor v on species s, and Z_iv_ represents the value of environmental variable v at site i. Detection probability (pi) was modeled as a function of camera-trap operational days, distance to water (dist_water), and camera type:

(4) $$logit(p_{sij})=\gamma_{0s}+\gamma_{1s}\;workday_{ij}+\gamma_{2s}\;dist\_water_{i}+\gamma_{3s}\;cam\_type_{i}$$

where p_sij_ represents the probability of detecting species s at site i during sampling occasion j, γ_0s_ is the intercept, and γ_1s_–γ_3s_ are regression coefficients describing the effects of detection covariates on detection probability. Here, workday_ij_ denotes the number of operational camera-trap days at site i during occasion j.

### 2.9. Clustering of Species Environmental Response Coefficients

To examine whether species could be grouped according to their environmental response patterns, we performed hierarchical clustering using species-specific environmental regression coefficients derived from the occupancy models. To reduce the influence of coefficient uncertainty on clustering results, these coefficients were subjected to uncertainty-weighted shrinkage prior to clustering. Specifically, each coefficient was multiplied by a shrinkage factor:

(5) $$E(\beta_{iv}|{\hat{\beta }}_{iv})={\hat{\beta }}_{iv}\cdot \frac{\tau^{2}}{\tau^{2}+\sigma_{iv}^{2}}$$

where ${\hat{\beta }}_{iv}$ is the posterior mean of the regression coefficient for species i and environmental predictor v, $\sigma_{iv}^{2}$ is the posterior variance of that coefficient, and $\tau^{2}$ is the overall variance of regression coefficients for predictor v across species. This procedure shrinks highly uncertain coefficients toward zero, thereby reducing the influence of statistical noise on multivariate clustering structure and allowing groups to better reflect stable ecological response patterns. Clustering was based only on occupancy-level environmental regression coefficients and did not include detection parameters. The shrinkage-adjusted beta coefficient matrix was first standardized, and pairwise distances among species were then defined as one minus the correlation coefficient. Hierarchical clustering was subsequently performed using the Ward method. Candidate clustering solutions were evaluated using mean silhouette width, and the optimal number of clusters was selected as the k value with the highest silhouette score. PERMANOVA was then performed on the corresponding distance matrix, and 9999 permutations were used to assess whether species groups occupied distinct regions of environmental response space.

### 2.10. Mantel Analysis

To compare species-level patterns derived from different data types, we calculated pairwise Jaccard distances among species based on site-level occurrence data, Bray–Curtis distances based on site-level RAI values, and distances based on environmental response coefficients (beta). Mantel tests with 9999 permutations were used to assess concordance among these species-level distance matrices.

All statistical analyses were conducted in R 4.5.2 [40], using the packages vegan (version 2.7-2) [39], betapart (version 1.6.1) [38], cluster (version 2.1.8.1) [41], and rstan (version 2.32.7) [42].

## 3. Results

**Figure 1:**
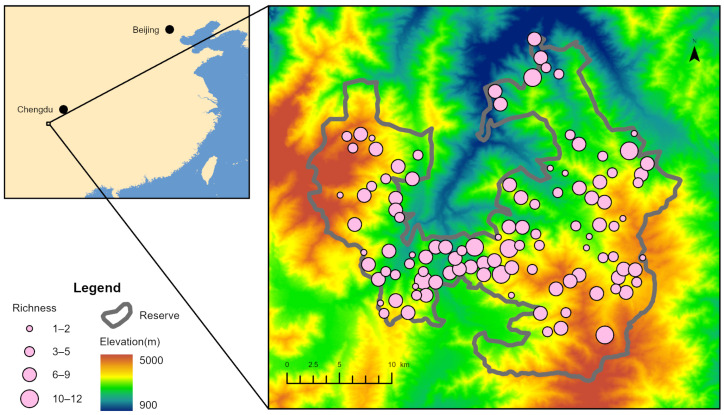
Spatial distribution of camera-trap sites and species richness in the study area. Circle size represents the number of species recorded by each camera trap. The background raster shows elevation, and the grey boundary outlines Liziping Nature Reserve.

A total of 109 camera-trap sites were deployed in this study, yielding 37,207 camera-trap days (Table S3) and 6688 independent detections. After excluding species with fewer than 30 independent detections and records from fewer than 10 camera-trap sites, 17 species were retained for analysis (Table S4). Species richness at individual camera-trap sites ranged from 1 to 12, with a mean of 5.7706 (Figure 1). Species richness varied among sites and showed a unimodal distribution with a slight right skew (Figure S2).

### 3.1. Community Beta Diversity

Beta-diversity decomposition showed that differences among communities were driven primarily by species turnover (mean turnover = 0.5354), whereas the nestedness component was relatively low (mean nestedness = 0.1862). Turnover accounted for 74.20% of total beta diversity, indicating that variation among sites was dominated by species replacement. To evaluate whether the observed level of community differentiation deviated from random expectations, we compared the observed mean pairwise Jaccard dissimilarity with a null distribution generated by the swap algorithm while preserving both site richness and species occurrence frequencies. The observed value did not differ significantly from the null expectation (observed = 0.7216, null mean = 0.7207, SES = 0.6818, *p* = 0.5207), suggesting that the overall magnitude of community differentiation was consistent with random expectations given site richness and species occurrence frequencies.

### 3.2. Species Similarity and Clustering

**Figure 2:**
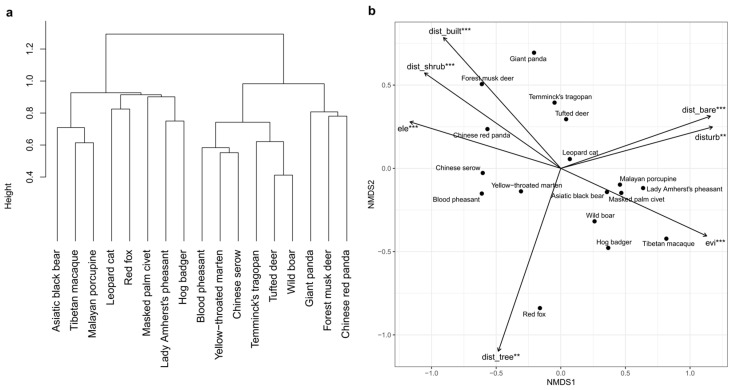
Species-level co-occurrence structure based on occurrence data. (**a**) Hierarchical clustering of species based on Jaccard dissimilarity calculated from species occurrence across camera-trap sites. (**b**) Non-metric multidimensional scaling (NMDS) ordination of species based on Jaccard dissimilarity. Points represent species in ordination space. Arrows indicate environmental variables fitted using envfit, with arrow length proportional to correlation strength (r^2^). Only significant variables (*p* < 0.05) are shown. Significance levels are indicated by asterisks (** *p* < 0.01, *** *p* < 0.001).

We further examined whether species co-occurrence patterns formed discrete modules. Hierarchical clustering analyses were conducted using Jaccard dissimilarity based on species occurrence across camera-trap sites (Figure 2a) and Bray–Curtis dissimilarity based on species-specific RAI values across sites (Figure S3a). Across the range of k = 2–16, mean silhouette values were consistently below 0.11, indicating weak separation among groups and providing little support for well-defined discrete modules. At the same time, Δ analysis (Figures S4 and S5) showed that the observed grouping structure differed significantly from random expectations at all k values (*p* < 0.01), indicating that similarity structure among species was non-random. Taken together with the low silhouette values, these results suggest that the observed structure is better interpreted as weak differentiation or continuous variation rather than strongly separated modular groups.

### 3.3. NMDS Ordination and Environmental Gradients

NMDS ordination further supported this interpretation, indicating that species co-occurrence and relative activity patterns were better characterized by continuous variation associated with environmental gradients than by clearly separated discrete modules. Two-dimensional ordinations based on Jaccard and Bray–Curtis dissimilarities both showed species distributed continuously in ordination space, with stress values of 0.2142 and 0.2139, respectively, suggesting gradual variation in co-occurrence relationships and relative activity structure among species (Figure 2b and Figure S3b). Environmental fitting analysis (Table S5) showed that environmental variables explained both ordination patterns consistently and relatively strongly. Elevation was the strongest correlate of species ordination structure, with r^2^ values of 0.3904 and 0.4036 for the Jaccard- and Bray–Curtis-based ordinations, respectively (both *p* < 0.001). Vegetation productivity (EVI) was the second strongest correlate, with r^2^ values of approximately 0.28 in both ordinations (both *p* < 0.001). Other variables significantly associated with species ordination structure included habitat variables, such as distance to bare land, built-up areas, shrubland, forest, and grassland, as well as recent anthropogenic disturbance, with r^2^ values ranging from 0.0551 to 0.1507 (all *p* < 0.05). In contrast, TWI, slope, northness, and distance to water showed relatively weak explanatory power (r^2^ = 0.0067–0.0429) and were non-significant in both ordinations (*p* > 0.05).

At the species level, NMDS ordinations based on Jaccard and Bray–Curtis dissimilarities showed partially concordant patterns (Figure 2b and Figure S3b). In both ordinations, species differentiation was structured primarily along gradients of elevation, vegetation productivity, habitat structure, and human disturbance, indicating that co-occurrence and relative activity patterns were both associated with the same broad set of environmental factors. Several species occupied broadly comparable positions in the two ordination spaces. For example, giant panda, forest musk deer (*Moschus berezovskii*), Chinese red panda (*Ailurus styani*), and Chinese serow (*Capricornis milneedwardsii*) were associated with the high-elevation and more remote side of the ordination space, whereas Tibetan macaque (*Macaca thibetana*) and Lady Amherst’s pheasant (*Chrysolophus amherstiae*) were positioned toward the higher-EVI side of the ordination space. However, a number of species showed noticeable shifts between the two ordinations, particularly along gradients related to habitat structure and disturbance. These differences suggest that Jaccard- and Bray–Curtis-based ordinations capture overlapping but not identical aspects of species differentiation, even though both support a continuous pattern of variation along environmental gradients.

### 3.4. Occupancy Analysis and Clustering Using Occupancy Coefficients

**Figure 3:**
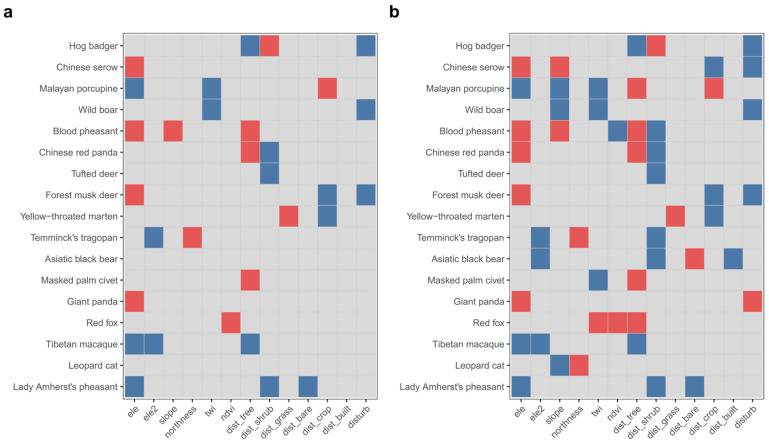
Species-specific environmental responses inferred from occupancy models. (**a**) Significant effects of environmental variables on occupancy probability, defined as regression coefficients with 90% credible intervals not overlapping zero. (**b**) Directional certainty of responses based on posterior direction probabilities (probability that β > 0); values > 0.9 indicate positive responses and values < 0.1 indicate negative responses. Red and blue cells indicate positive and negative effects, respectively, whereas grey cells indicate non-significant or uncertain responses. Rows correspond to species and columns to environmental variables. The heterogeneous pattern across species indicates substantial differentiation in environmental responses within the community.

The occupancy model converged well, with all environmental variable parameters having Rhat values below 1.001, indicating stable estimation. Of the 17 species, all but the Asiatic black bear and leopard cat showed significant effects for at least one environmental variable (Figure 3). Although some species did not meet traditional significance thresholds, posterior probabilities of the direction of effects indicated that all species showed clear directional responses to at least one environmental variable (posterior probability > 0.9 or <0.1), suggesting distinct environmental response differentiation.

Elevation-related variables had the strongest influence on species differentiation. Standardized elevation (ele) and its squared term (ele2) revealed different response patterns, such as low elevation preference, high elevation preference, and hump-shaped responses at mid-elevation. Habitat structure and human activity variables also showed species-specific effects, while aspect, NDVI, distance to built-up areas, distance to grassland, and distance to bare land had clear directional effects only for some species. These findings suggest ecological niche differentiation rather than a single, consistent environmental filtering pattern.

Specifically, in the study region, Lady Amherst’s pheasant showed a negative linear response to elevation, indicating higher occupancy toward lower elevations, whereas Tibetan macaque showed a hump-shaped response with an optimum at relatively low elevations. In contrast, Chinese serow and blood pheasant (*Ithaginis cruentus*) showed a monotonic increases in occupancy with elevation, while Asiatic black bear (*Ursus thibetanus*) and Temminck’s tragopan (*Tragopan temminckii*) both displayed hump-shaped responses with optima at mid-elevations. For habitat structure variables, Temminck’s tragopan and tufted deer were associated with sites closer to shrubland, whereas hog badger (*Arctonyx collaris*) showed the opposite response, being associated with sites farther from shrubland and closer to forest. Chinese red panda and blood pheasant were associated with sites closer to forest and shrubland. Regarding human-related variables, distance to built-up areas had a negative effect on Asiatic black bear, while recent disturbances such as livestock grazing had a positive effect on giant panda, but negative effects on Chinese serow, forest musk deer, hog badger, and wild boar.

**Figure 4:**
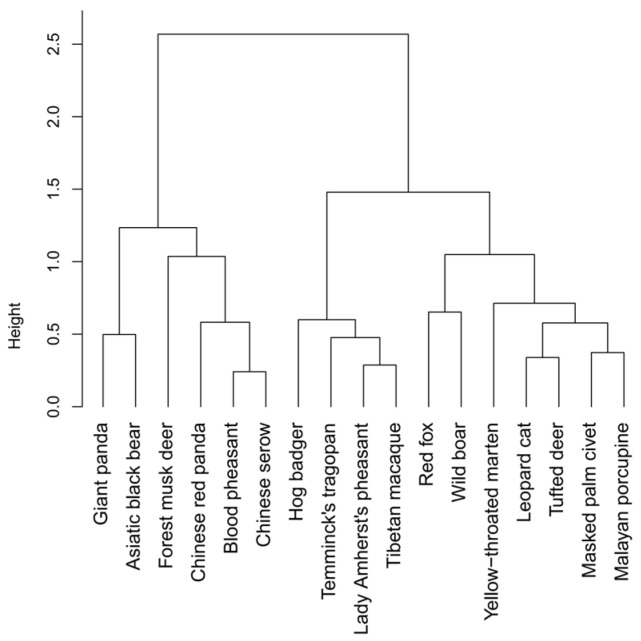
Hierarchical clustering of species based on shrinkage-adjusted environmental regression coefficients from occupancy models.

Hierarchical clustering based on shrinkage-adjusted species-specific environmental regression coefficients showed that mean silhouette width was highest at k = 2 (Figure S6), with a value of 0.3486, indicating that a two-cluster solution provided the best separation among the candidate groupings (Figure 4). PERMANOVA further suggested that these two clusters occupied distinct regions of multivariate environmental response space (r^2^ = 0.4307, F = 11.3463, *p* < 0.001), indicating that the identified groups corresponded to different environmental response patterns. The first cluster included giant panda, Asiatic black bear, forest musk deer, Chinese red panda, blood pheasant, and Chinese serow, whereas the second cluster included hog badger, Temminck’s tragopan, Lady Amherst’s pheasant, Tibetan macaque, red fox (*Vulpes vulpes*), wild boar (*Sus scrofa*), yellow-throated marten (*Martes flavigula*), leopard cat (*Prionailurus bengalensis*), tufted deer (*Elaphodus cephalophus*), masked palm civet (*Paguma larvata*), and Malayan porcupine (*Hystrix brachyura*).

### 3.5. Mantel Test on Species Co-Occurrence and Environmental Response

Mantel tests showed no significant correlation between Jaccard distances based on presence–absence data and Bray–Curtis distances based on species-specific RAI values (r = 0.0678, *p* = 0.2907). In contrast, Jaccard distances were positively correlated with distances derived from shrinkage-adjusted environmental response coefficients (r = 0.2529, *p* = 0.0115), suggesting partial concordance between species co-occurrence structure and similarity in environmental responses. Bray–Curtis distances were not significantly correlated with beta distances (r = −0.0408, *p* = 0.6421), suggesting that relative activity structure did not vary in parallel with environmental response differentiation.

## 4. Discussion

Understanding the spatial organization of communities within protected areas and its environmental drivers is important for elucidating the mechanisms that maintain biodiversity and for improving conservation management [16,43]. This study shows that the mammal and galliform bird communities in Liziping Nature Reserve do not exhibit distinct boundary-separated structures, but instead resemble a continuous gradient pattern shaped by environmental factors. Beta diversity was primarily driven by species turnover, indicating that the community differences between sites were largely due to species replacement rather than simple nestedness. Clustering analyses based on species co-occurrence and relative activity intensity did not identify well-separated species modules. Furthermore, NMDS ordination revealed that both species co-occurrence and relative activity structures changed continuously along environmental gradients. Together, these results suggest that the communities in the study area are not composed of clearly distinct, discrete units but are more likely the result of continuous environmental selection processes [44,45]. Occupancy models further indicated that species responded differently to environmental gradients, suggesting that coexistence in this area is more reliant on niche differentiation and environmental heterogeneity than on simple discrete partitioning. Taken together, the findings suggest that the community in Liziping Nature Reserve is better described as a continuous gradient structure rather than a discrete modular one. Environmental heterogeneity and the niche differentiation it promotes are likely key mechanisms maintaining multispecies coexistence in this reserve.

### 4.1. Environmental Gradients and Community Turnover

The turnover-dominated pattern observed in Liziping Nature Reserve indicates substantial environmental heterogeneity within the reserve. When considered together with the ordination and environmental fitting results, elevation emerged as the primary factor structuring species association patterns in the study area, consistent with a broad body of research on mountain communities [46,47]. Vegetation productivity, habitat structure, and human-related variables also contributed to continuous environmental variation within the reserve. Because species differ in their environmental tolerances and resource-use patterns, different sites are more likely to support distinct species assemblages, leading to continuous species replacement along environmental gradients rather than simple gains or losses of species. Such turnover may help maintain community diversity within protected areas [48,49].

The null model results further suggest that this turnover pattern does not imply strong discrete community partitioning within the reserve, but is more likely the outcome of the combined effects of species occurrence frequencies, site-level richness, and environmental gradients. This finding also implies that conservation management should not focus solely on local richness hotspots, but should also prioritize the environmental heterogeneity maintained by elevational, habitat, and disturbance gradients. Avoiding landscape homogenization or overly uniform management may therefore be important for preserving the diverse habitat conditions required by different species [50,51].

### 4.2. Species-Specific Environmental Responses and Niche Differentiation

The occupancy model results indicate that species in the study area do not follow a single, consistent pattern of environmental filtering; instead, they exhibit pronounced species-specific environmental responses. Species responded differently to elevation and habitat structure, suggesting that coexistence is more likely maintained through differentiated environmental preferences and resource-use strategies within a shared landscape [52,53]. For example, species exhibited contrasting responses along the elevational gradient, including low-elevation preferences, high-elevations preferences, and both symmetrical and skewed hump-shaped responses. Responses to habitat structure variables were likewise inconsistent or even opposite among species. These patterns suggest that multispecies coexistence in the reserve is more likely supported by niche differentiation under environmentally heterogeneous conditions rather than by a uniform environmental filtering process.

This result is broadly consistent with the general patterns reported in previous studies of habitat selection by mountain mammals and ground-dwelling birds. Existing studies have shown that elevation, vegetation conditions, and habitat structure are key determinants of wildlife distribution in mountain systems, but that the direction and relative importance of these effects often differ markedly among species. Among ungulates, for example, tufted deer are typically associated with forest habitats characterized by relatively high vegetation productivity and good cover [54], forest musk deer are often linked to higher elevations, greater vegetation cover, and better forest conditions [55], and Chinese serow tend to prefer mountain habitats with denser vegetation cover and relatively stable climatic conditions [5,56,57]. Likewise, the spatial distributions of galliform birds are often influenced by forest cover, shrub–grassland mosaics, and microhabitat structure. Temminck’s tragopan, for instance, is reported to depend more strongly on forest microhabitats with complex understory structure and proximity to streams [58], whereas blood pheasant has been associated more closely with high-elevation forest–open habitat ecotones and fragmented landscapes [59]. These previous findings are generally consistent with our results, in which different species showed low-elevation preferences, high-elevation preferences, or mid-elevation optima along the elevational gradient, and also differed in their responses to habitat-structure variables related to forest, shrubland, grassland, and bare land. Overall, these results indicate that multispecies communities do not respond uniformly to a single environmental gradient; rather, coexistence within the reserve is more likely achieved through differentiated habitat selection among species.

It is also notable that the proportion of environmental effects reaching conventional significance thresholds in the occupancy models was relatively limited, whereas directional differentiation among species was widespread. Clustering based on shrinkage-adjusted β coefficients further showed that species could be divided into two broad groups with contrasting environmental response tendencies, indicating substantial heterogeneity in ecological responses within the community. Rather than implying sharply separated ecological modules, these results are better interpreted as evidence that species differ consistently in the direction and combination of their responses to environmental gradients. Together with the ordination results, this supports the view that community structure in the reserve reflects continuous but non-random ecological differentiation among species.

### 4.3. Multiple Ecological Dimensions Revealed by Different Data Types

Mantel tests further indicated that species relationships inferred from different data types were not entirely consistent. Jaccard distances were modestly but significantly correlated with distances derived from environmental response coefficients, whereas Bray–Curtis distances were not correlated with either of them. This suggests that the species co-occurrence structure characterized by presence–absence data is partially consistent with patterns of environmental response differentiation, whereas similarity based on relative activity intensity reflects a partially independent ecological dimension.

In other words, the environmental response patterns that determine whether a species can occur at a given site do not necessarily determine how intensively it uses that site once present. Because both the occupancy model and the Jaccard analysis are based on presence–absence data, occurrence information can effectively reveal environmental suitability and broad distribution patterns [60], but may not fully capture the degree to which species actually use available habitats [61]. Consequently, when evaluating habitat quality and conservation priorities, relying solely on occurrence data may lead to underestimating the complexity of community organization, and complementary information on habitat use or activity intensity should also be considered.

### 4.4. Human Disturbance

Although natural environmental gradients remained the primary drivers of community structure, the influence of human disturbance should not be overlooked. In this study, distance to long-term landscape modification and recent disturbance intensity represented two different types of anthropogenic pressure. The former mainly reflects long-term landscape modification associated with roads, settlements, and agricultural land, which occurs primarily near the reserve’s boundaries. In contrast, recent disturbances represent short-term or recurrent human activities, such as livestock grazing and other human presence. These disturbances can penetrate more diffusely into the interior of the reserve and influence local habitat conditions and species activity at finer spatial scales. The relatively weak correlation between these two variables (Figure S1) suggests that different types of human disturbance may operate through largely independent spatial distributions and ecological mechanisms.

Combining the co-occurrence ordination and occupancy results shows that species responded differently to these two broad types of disturbance. In this study, distance to built-up areas and distance to cropland primarily reflect long-term landscape modification and land-use intensity associated with roads, settlements, and agricultural activities, whereas recent disturbance more directly represents recurrent short-term human activities such as livestock grazing and human presence. The NMDS ordinations showed that all three variables were significantly associated with community differentiation, indicating that both long-term landscape modification and short-term human activities contributed to shaping community structure. However, species positions along these disturbance axes were only partially concordant between the Jaccard- and Bray–Curtis-based ordinations, suggesting that human influence did not affect species co-occurrence patterns and relative activity structure in exactly the same way. In other words, human disturbance should not be treated as a single axis of influence [9,10], but rather as a set of pressures operating at different spatial scales and over different durations.

At the species level, occupancy analysis further showed that the direction of species responses to these human influences was not consistent. Recent disturbance had a positive effect on giant panda, but negative effects on Chinese serow, forest musk deer, hog badger, and wild boar, indicating that recurrent human activities such as livestock grazing do not affect all species in the same way. These patterns indicate that human activities still exert clear suppressive effects on some species.

Previous studies have reported that livestock grazing can pose substantial threats to wildlife such as giant pandas in some reserves [62,63], although other studies suggest that livestock presence does not necessarily have significant effects on species such as giant pandas, Chinese serows, or forest musk deer [63]. In the present study, giant pandas did not show a clear negative response to recent disturbance. One possible explanation is that human activities within panda habitats in Liziping Nature Reserve have been relatively well managed during the study period, so disturbance intensity may not have reached the threshold required to trigger strong avoidance behavior. In contrast, distance to built-up areas showed a clear negative effect only on Asiatic black bear, indicating that strong responses to built landscapes were not widespread across species and may depend more on species ecology and the spatial scale of disturbance. The results for the distance from cropland gradient are also noteworthy. Chinese serow, forest musk deer, and yellow-throated marten tended to occur closer to cropland, whereas Malayan porcupine tended to occur farther from it. This does not necessarily mean that cropland itself is attractive to these species. Rather, it may suggest that under the current management regime of the reserve, agricultural activities and their associated human influences have not spread broadly into surrounding natural habitats at larger spatial scales, and that their effects remain more localized around habitat edges. In other words, distance to cropland reflects not only the presence of cropland itself, but also the combined effects of edge vegetation conditions, the spatial extent of disturbance spillover, and species-specific trade-offs between resources and risk.

These interspecific differences are consistent with the general pattern reported in previous studies, namely that wildlife responses to human influence often depend on disturbance type, spatial scale, and species-specific ecological traits. For forest-dependent species, for example, forest musk deer are generally reported to avoid cropland and built landscapes [64], while habitat quality for Chinese red panda declines with increasing roads, settlements, and human footprint [65], indicating that long-term landscape modification can progressively compress suitable habitat for disturbance-sensitive species. Yellow-throated marten also tends to occur in forests farther from areas of human use [66], which is consistent with the broader view that increasing human land use may reduce the integrity of forest habitat. On the other hand, some mesocarnivores and generalist species often show weaker avoidance of long-term landscape disturbance and may even maintain relatively high activity in edge habitats. For example, leopard cats have shown some tolerance of immediate human activity in certain regions [66,67], and red foxes often remain active near habitat edges and built environments [68]. The case of Asiatic black bear further illustrates that even within a single species, responses to human influence may differ across spatial scales: at local scales, bears may come into frequent contact with people and generate conflicts [69], whereas at broader scales, human landscape expansion can still substantially reduce suitable habitat [70].

Therefore, the pattern revealed by this study is not that human disturbance exerts a uniformly negative effect on all species. Rather, long-term landscape modification and recent activity disturbance represent different dimensions of human influence, differing in spatial distribution, mechanisms of action, and ecological consequences. From a conservation perspective, this means that built-up areas, cropland, and recent human activities should not be treated as a single form of pressure; instead, their spatial extent, pathways of influence, and potential consequences for different species should be evaluated separately.

## 5. Conclusions

Overall, mammal and galliform bird communities in Liziping Nature Reserve did not form clearly bounded discrete modules, but were instead primarily organized as continuous variation along environmental gradients, with community differences driven mainly by species turnover rather than nestedness. The pronounced differentiation in species-specific environmental responses suggests that multispecies coexistence in the study area is more likely maintained by niche differentiation under environmentally heterogeneous conditions than by fixed community modules or spatial segregation. These findings highlight the importance of maintaining environmental gradients, habitat connectivity [71], and microhabitat diversity in order to better encompass broader community patterns and multispecies conservation needs within the reserve. Although human activities did not produce a uniformly strong negative effect at the community level in the present study, they had clear effects on some species, indicating that continued regulation of livestock grazing [72] and control of the cumulative impacts of human disturbance remain necessary.

Under the long-standing conservation framework of Liziping Nature Reserve, giant panda and its habitat have remained the core management priority, a focus that has had clear scientific and practical value and has contributed substantially to habitat protection, patrolling, and ecological restoration. Our results suggest that, while maintaining these strengths, the reserve’s conservation objectives should be further expanded to include the protection of biological communities, ecosystems, and ecological processes across environmental gradients, thereby moving from a single flagship-species focus toward a more integrated “species–community–ecosystem–ecological process” approach. At the same time, these findings should be interpreted in the context of ongoing and not fully eliminated human activities within the reserve. Although patrolling, public outreach, and alternative livelihood measures have reduced grazing pressure to some extent, livestock and other human activities still persist in parts of the reserve. This suggests that future management should place greater emphasis on spatially and seasonally differentiated regulation of grazing, farming, and tourism, especially in habitats and species distribution areas that are more sensitive to disturbance. In forest zones, management should prioritize the control of road disturbance and engineering activities, together with stricter constraints on collection and grazing. In shrubland and meadow zones, priority should be given to controlling grazing intensity and reducing the impacts of seasonal use and habitat fragmentation. In edge areas near surrounding human activities, management should strengthen community co-management, ecological restoration, and disturbance buffering, while also optimizing farming practices, establishing buffer zones, limiting edge expansion, and regulating tourist routes and timing to reduce the spread of cropland- and tourism-related disturbance into adjacent natural habitats. The revised Regulations of the People’s Republic of China on Nature Reserves, which came into force in 2026, explicitly allow necessary production and living activities of original residents provided that their existing scale and intensity are not expanded. Within this institutional framework, reserve management should therefore further promote zoned and differentiated regulation, with particular attention to preventing increases in disturbance intensity and its spread into key habitats and areas occupied by disturbance-sensitive species, thereby strengthening not only giant panda conservation but also the protection of overall biodiversity, ecological integrity, and resilience.

## Data Availability

Data will be made available upon request.

## Supplementary Materials

The following supporting information can be downloaded at https://www.mdpi.com/article/10.3390/biology15090672/s1, Figure S1: Pairwise correlations among environmental variables; Figure S2: Distribution of species richness recorded across camera-trap sites. Species richness represents the number of species detected at each camera station during the survey period; Figure S3: Species-level clustering and ordination based on Bray–Curtis dissimilarity. (a) Hierarchical clustering of species based on Bray–Curtis dissimilarity calculated from species-specific RAI values across camera-trap sites. Clustering was performed using Ward’s method (ward.D2). (b) Non-metric multidimensional scaling (NMDS) ordination of species based on Bray–Curtis dissimilarity. Points represent species in ordination space. Arrows indicate environmental variables fitted using envfit, with arrow length proportional to correlation strength (r^2^). Only variables significantly associated with community composition (*p* < 0.05) are shown. Significance levels are indicated by asterisks (* *p* < 0.05, ** *p* < 0.01, *** *p* < 0.001); Figure S4: Permutation tests of Δ (between-group minus within-group distance) for hierarchical clustering based on Jaccard dissimilarity. Histograms represent the null distribution obtained by randomly permuting group labels (9999 permutations), and red lines indicate the observed Δ values for each cluster number (k = 2–14); Figure S5: Permutation tests of Δ (between-group minus within-group distance) for hierarchical clustering based on Bray–Curtis dissimilarity. Histograms represent the null distribution obtained by randomly permuting group labels (9999 permutations), and red lines indicate the observed Δ values for each cluster number (k = 2–14); Figure S6: Permutation tests of Δ based on distances among species’ occupancy model coefficients (β of ψ). Histograms show the null distributions from 9999 permutations, and red lines indicate the observed Δ for clustering solutions (k = 2–14); Table S1: Variance inflation factors (VIF) for environmental variables retained in the final models; Table S2: Interpretation of standardized elevation effects in the occupancy model. Elevation (ele) was standardized prior to modeling, and the quadratic term (ele^2^) was calculated as the square of the standardized elevation. When ele^2^ < 0, the response curve is concave (∩-shaped) with a potential optimum elevation. When ele^2^ > 0, the curve is convex (∪-shaped) with a potential minimum at intermediate elevations. The sign of ele determines the direction or skewness of the curve along the elevation gradient; Table S3. Camera-trap days for each camera-trap site. Table S4: Camera-trap records of the 17 species retained for analysis; Table S5: Correlations between environmental variables and NMDS ordinations based on Bray–Curtis and Jaccard dissimilarities, assessed using envfit. r^2^ indicates the strength of the relationship and *p* values are from permutation tests.
