# Supplementary material for: Environmental Gradients Shape Mammal and Galliform Bird Communities in a Mountain Reserve Through Species Turnover and Niche Differentiation
Source: Biology (Basel). 2026 Apr 24;15(9):672. doi: 10.3390/biology15090672 (PMC13162646; doi:10.3390/biology15090672)
Supplement: Supplementary file 1 [file biology-15-00672-s001.zip › biology-4231431-supplementary.pdf]

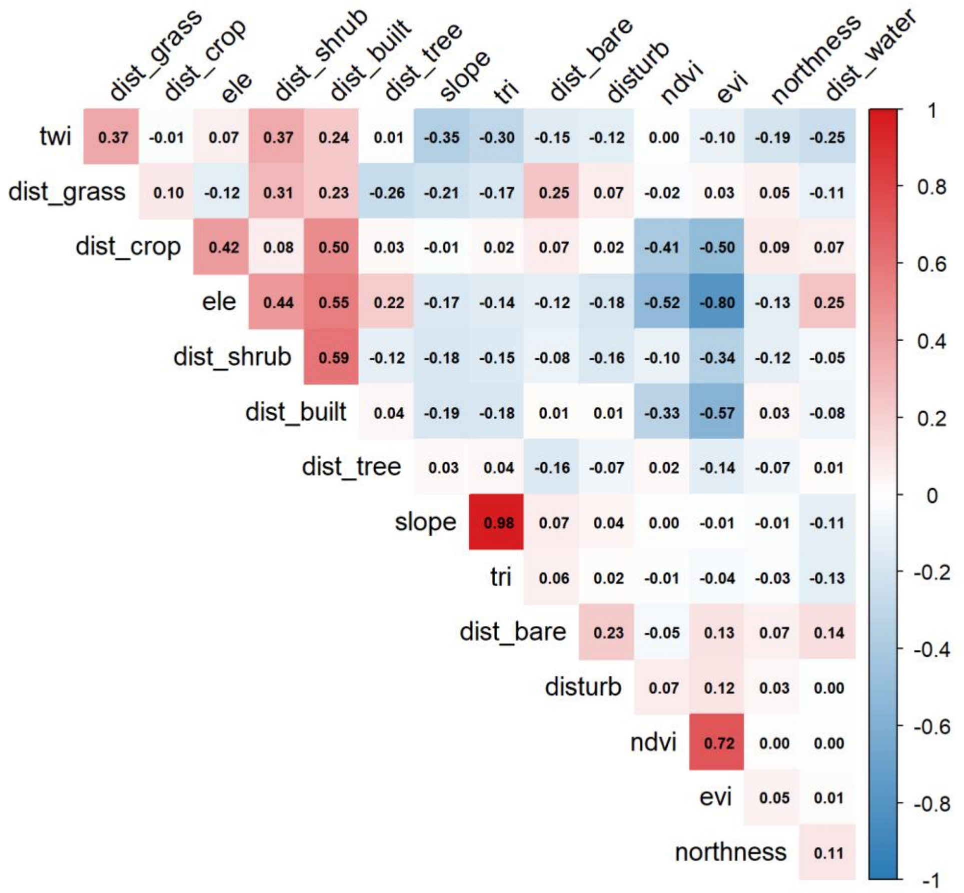

Figure S1. Pairwise correlations among environmental variables.

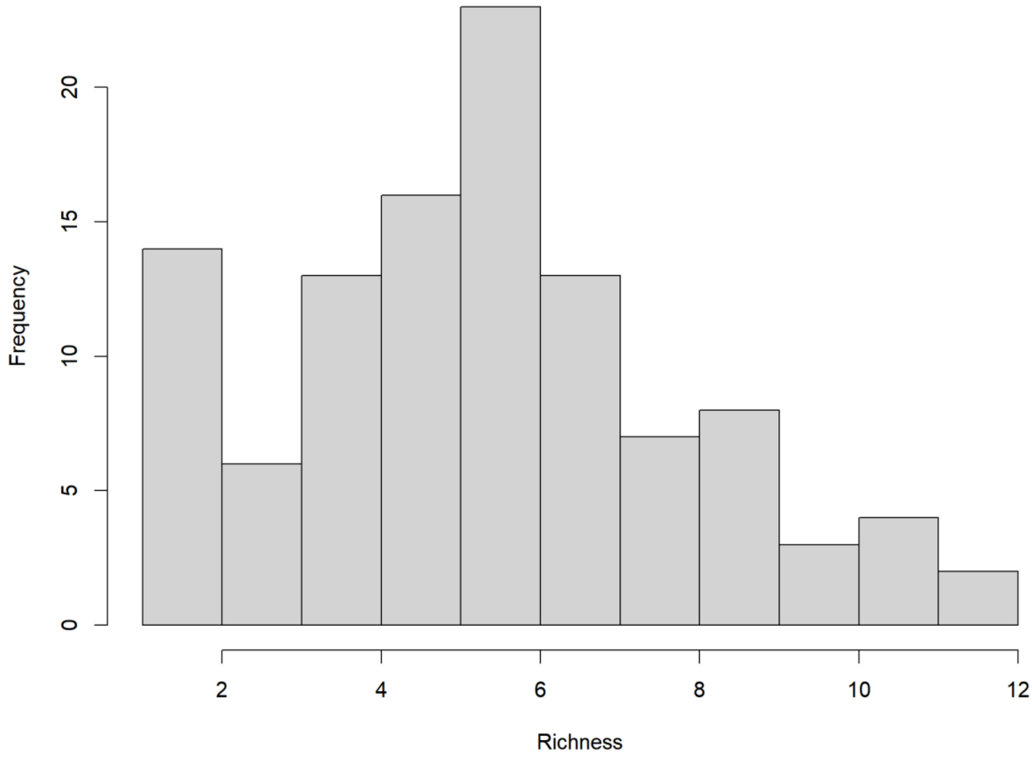

Figure S2. Distribution of species richness recorded across camera-trap sites. Species richness represents the number of species detected at each camera station during the survey period.

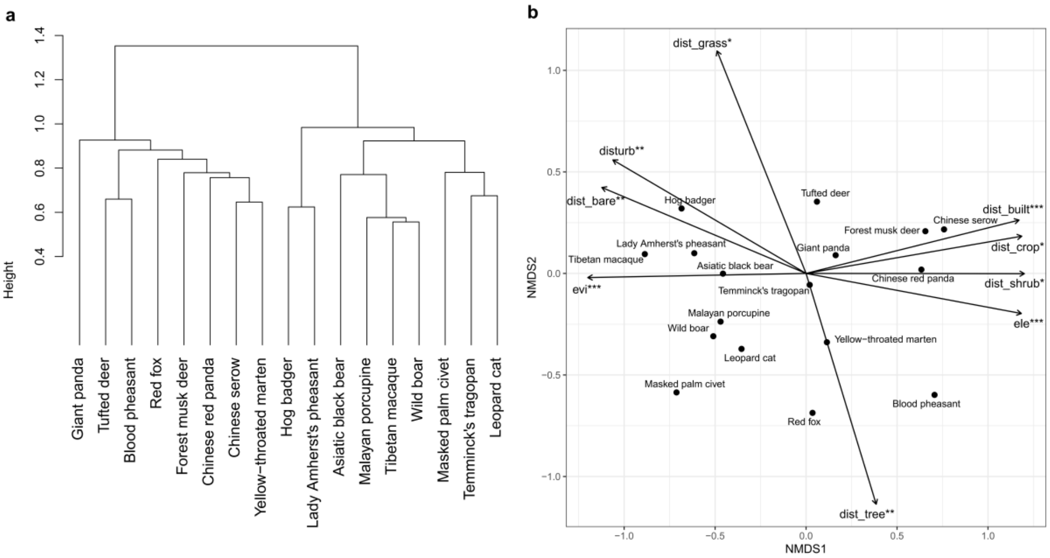

Figure S3. Species-level clustering and ordination based on Bray–Curtis dissimilarity. (a) Hierarchical clustering of species based on Bray–Curtis dissimilarity calculated from species-specific RAI values across camera-trap sites. Clustering was performed using Ward's method (ward.D2). (b) Non-metric multidimensional scaling (NMDS) ordination of species based on Bray–Curtis dissimilarity. Points represent species in ordination space. Arrows indicate environmental variables fitted using envfit, with arrow length proportional to correlation strength(r^2). Only variables significantly associated with community composition ($p < 0.05$) are shown. Significance levels are indicated by asterisks (*$p < 0.05$, **$p < 0.01$, ***$p < 0.001$).

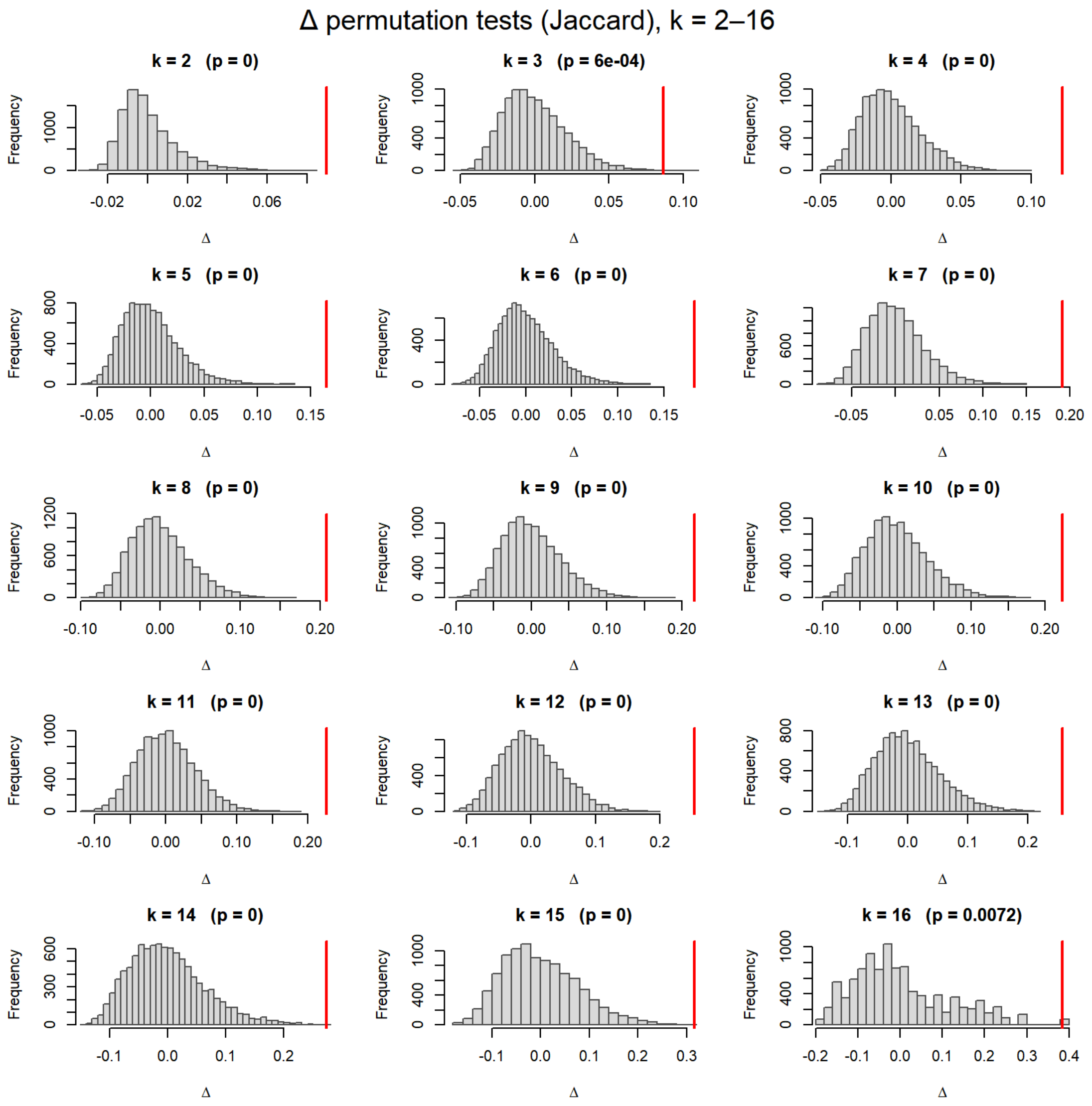

Figure S4. Permutation tests of Δ (between-group minus within-group distance) for hierarchical clustering based on Jaccard dissimilarity. Histograms represent the null distribution obtained by randomly permuting group labels (9,999 permutations), and red lines indicate the observed Δ values for each cluster number (k = 2–14).

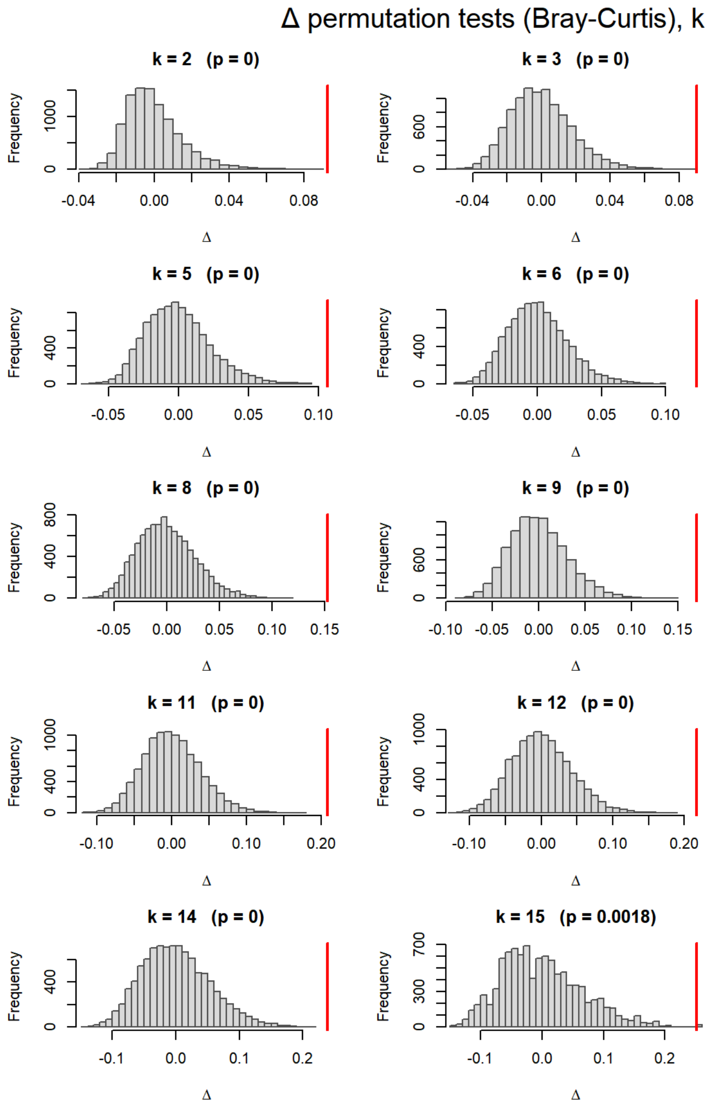

Figure S5. Permutation tests of Δ (between-group minus within-group distance) for hierarchical clustering based on Bray-Curtis dissimilarity. Histograms represent the null distribution obtained by randomly permuting group labels (9,999 permutations), and red lines indicate the observed Δ values for each cluster number (k = 2–14).

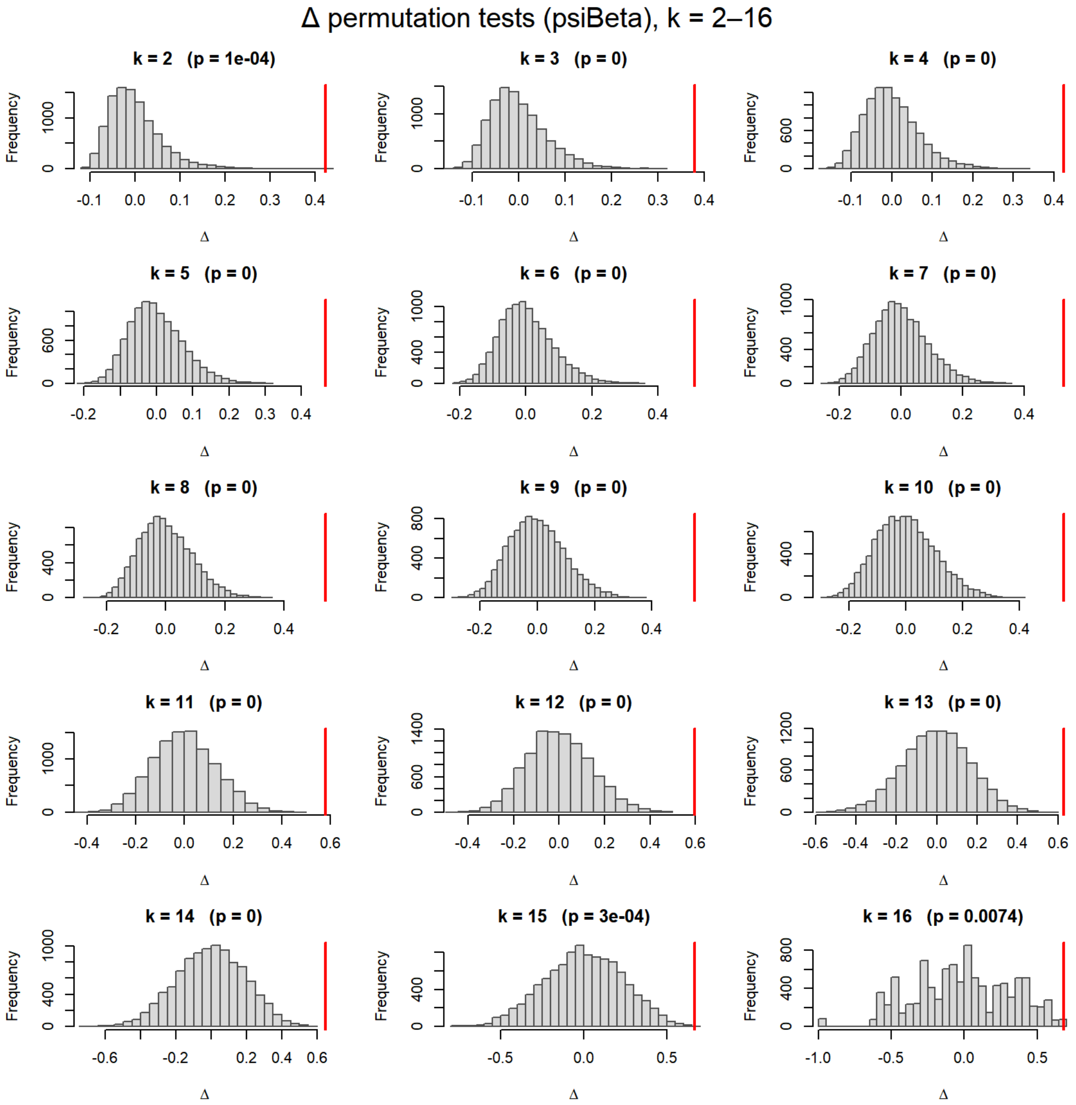

Figure S6. Permutation tests of *Δ* based on distances among species' occupancy model coefficients (β of ψ). Histograms show the null distributions from 9,999 permutations, and red lines indicate the observed *Δ* for clustering solutions (k = 2–14).

Table S1 Variance inflation factors (VIF) for environmental variables retained in the final models.

Variable	VIF
ele	3.2649
dist_built	2.7142
dist_shrub	2.4735
dist_crop	1.7174
ndvi	1.6621
dist_grass	1.6531
twi	1.6282
dist_water	1.3897
slope	1.2802
dist_bare	1.2470
dist_tree	1.2446
disturb	1.1456
northness	1.1456

Table S2. Interpretation of standardized elevation effects in the occupancy model. Elevation (ele) was standardized prior to modeling, and the quadratic term (ele^2) was calculated as the square of the standardized elevation. When **$ele^2 < 0$**, the response curve is concave (∩-shaped) with a potential optimum elevation. When **$ele^2 > 0$**, the curve is convex (∪-shaped) with a potential minimum at intermediate elevations. The sign of **ele** determines the direction or skewness of the curve along the elevation gradient.

ele	ele^2	Curve shape	Ecological interpretation (relative elevation gradient)
0	0	Flat	No evident elevation effect on occupancy
+	0	Linear increase	Occupancy increases with elevation
−	0	Linear decrease	Occupancy increases toward lower elevations
0	−	Symmetrical hump-shaped	Mid-elevation optimum; lower occupancy at both low and high elevations
0	+	Symmetrical U-shaped	Higher occupancy at both low and high elevations; lower in the middle
+	−	Skewed hump-shaped	Elevation optimum present, with the peak shifted toward higher elevations
−	−	Skewed hump-shaped	Elevation optimum present, with the peak shifted toward lower elevations
+	+	Upward-opening J/U shape	Occupancy increases more strongly at higher elevations
−	+	Reverse J/U shape	Occupancy higher at lower elevations and decreases toward higher elevations

Table S3. Camera-trap days for each camera-trap site.

Camera ID	Camera days	Camera ID	Camera days	Camera ID	Camera days
**1**	244	**38**	188	**75**	383
**2**	411	**39**	377	**76**	610
**3**	410	**40**	509	**77**	236
**4**	249	**41**	371	**78**	213
**5**	411	**42**	753	**79**	346
**6**	475	**43**	822	**80**	189
**7**	216	**44**	827	**81**	176
**8**	299	**45**	195	**82**	331
**9**	243	**46**	808	**83**	218
**10**	411	**47**	352	**84**	214
**11**	367	**48**	188	**85**	242
**12**	409	**49**	302	**86**	258
**13**	231	**50**	195	**87**	323
**14**	207	**51**	946	**88**	432
**15**	518	**52**	536	**89**	224
**16**	225	**53**	332	**90**	217
**17**	232	**54**	200	**91**	236
**18**	234	**55**	158	**92**	509
**19**	256	**56**	460	**93**	546
**20**	244	**57**	332	**94**	203
**21**	164	**58**	263	**95**	148
**22**	233	**59**	588	**96**	248
**23**	191	**60**	408	**97**	331
**24**	161	**61**	570	**98**	313
**25**	598	**62**	167	**99**	183
**26**	309	**63**	183	**100**	395
**27**	572	**64**	710	**101**	332
**28**	166	**65**	370	**102**	220
**29**	353	**66**	918	**103**	188
**30**	184	**67**	388	**104**	319
**31**	191	**68**	243	**105**	331
**32**	167	**69**	209	**106**	213
**33**	942	**70**	124	**107**	242
**34**	240	**71**	176	**108**	506
**35**	491	**72**	578	**109**	219
**36**	161	**73**	137		
**37**	435	**74**	180		

Table S4. Camera-trap records of the 17 species retained for analysis.

English name	Scientific name	Independent detections
Tibetan macaque	*Macaca thibetana*	206
Chinese serow	*Capricornis milneedwardsii*	217
Forest musk deer	*Moschus berezovskii*	46
Tufted deer	*Elaphodus cephalophus*	1792
Malayan Porcupine	*Hystrix brachyura*	250
Wild boar	*Sus scrofa*	371
Leopard cat	*Prionailurus bengalensis*	50
Red fox	*Vulpes vulpes*	116
Giant panda	*Ailuropoda melanoleuca*	35
Chinese Red Panda	*Ailurus styani*	118
Asiatic black bear	*Ursus thibetanus*	53
Yellow-throated marten	*Martes flavigula*	160
Masked palm civet	*Paguma larvata*	51
Hog badger	*Arctonyx collaris*	80
Lady Amherst's pheasant	*Chrysolophus amherstiae*	78
Temminck’s tragopan	*Tragopan temminckii*	187
Blood pheasant	*Ithaginis cruentus*	710

Table S5. Correlations between environmental variables and NMDS ordinations based on Bray–Curtis and Jaccard dissimilarities, assessed using envfit. r^2 indicates the strength of the relationship and p values are from permutation tests.

Variable	Jaccard		Bray-Curtis	
	r^2	p	r^2	p
ele	0.3904	0.0001	0.4036	0.0001
evi	0.2881	0.0001	0.2872	0.0001
dist_bare	0.1452	0.0005	0.1048	0.0032
dist_built	0.1268	0.0005	0.1507	0.0003
dist_shrub	0.1208	0.0010	0.0840	0.0104
dist_tree	0.1042	0.0034	0.1070	0.0031
disturb	0.1060	0.0034	0.0900	0.0069
dist_grass	0.0551	0.0485	0.0638	0.0273
dist_crop	0.0430	0.0926	0.0741	0.0149
twi	0.0429	0.0987	0.0191	0.3548
dist_water	0.0281	0.2222	0.0277	0.2255
northness	0.0266	0.2375	0.0108	0.5561
slope	0.0067	0.6988	0.0103	0.5825
